# Dynamics behind the scale up of evidence-based obesity prevention: protocol for a multi-site case study of an electronic implementation monitoring system in health promotion practice

**DOI:** 10.1186/s13012-017-0686-5

**Published:** 2017-12-06

**Authors:** Kathleen P. Conte, Sisse Groen, Victoria Loblay, Amanda Green, Andrew Milat, Lina Persson, Christine Innes-Hughes, Jo Mitchell, Sarah Thackway, Mandy Williams, Penelope Hawe

**Affiliations:** 1 Menzies Centre for Health Policy, School of Public Health & The Australian Prevention Partnership Centre, University of Sydney, Level 6, Charles Perkins Centre, D17, Sydney, NSW 2006 Australia; 2 NSW Office of Preventive Health, Liverpool Hospital, Locked Bag 7103, Liverpool BC, NSW 1871 Australia; 3 Centre for Epidemiology and Evidence, New South Wales Ministry of Health, 73 Miller Street, North Sydney, NSW 2060 Australia; 4 Centre for Population Health, New South Wales Ministry of Health, 73 Miller Street, North Sydney, NSW 2060 Australia; 5 South Western Sydney Local Health District, Locked Mail Bag 7279, Liverpool BC, NSW 1871 Australia

**Keywords:** Implementation science, Performance monitoring, Prevention, Partnership research, Key performance indicators, Ethnography, Scale-up

## Abstract

**Background:**

The effectiveness of many interventions to promote health and prevent disease has been well established. The imperative has therefore shifted from amassing evidence about efficacy to scale-up to maximise population-level health gains. Electronic implementation monitoring, or ‘e-monitoring’, systems have been designed to assist and track the delivery of preventive policies and programs. However, there is little evidence on whether e-monitoring systems improve the dissemination, adoption, and ongoing delivery of evidence-based preventive programs. Also, given considerable difficulties with e-monitoring systems in the clinical sector, scholars have called for a more sophisticated re-examination of e-monitoring’s role in enhancing implementation.

**Methods:**

In the state of New South Wales (NSW), Australia, the Population Health Information Management System (PHIMS) was created to support the dissemination of obesity prevention programs to 6000 childcare centres and elementary schools across all 15 local health districts. We have established a three-way university-policymaker-practice research partnership to investigate the impact of PHIMS on practice, how PHIMS is used, and how achievement of key performance indicators of program adoption may be associated with local contextual factors. Our methods encompass ethnographic observation, key informant interviews and participatory workshops for data interpretation at a state and local level. We use an on-line social network analysis of the collaborative relationships across local health district health promotion teams to explore the relationship between PHIMS use and the organisational structure of practice.

**Discussion:**

Insights will be sensitised by institutional theory, practice theory and complex adaptive system thinking, among other theories which make sense of socio-technical action. Our working hypothesis is that the science of getting evidence-based programs into practice rests on an in-depth understanding of the role they play in the on-going system of local relationships and multiple accountabilities. Data will be synthesised to produce a typology to characterise local context, PHIMS use and key performance indicator achievement (of program implementation) across the 15 local health districts. Results could be used to continuously align e-monitoring technologies within quality improvement processes to ensure that such technologies enhance practice and innovation. A partnership approach to knowledge production increases the likelihood that findings will be put into practice.

## Background

The effectiveness of many interventions aimed at promoting health and preventing disease has been well established [1]. The imperative has thus shifted from amassing evidence of efficacy to delivering interventions at scale to achieve maximum population-level health gains [2]. Governments, funders and organisations working in the preventive health sphere are increasingly developing electronic implementation monitoring, or ‘e-monitoring’, systems to track the distribution of prevention policies, activities and programs. Some organisations contract commercial software companies to tailor existing software programs to their needs, while others create their own bespoke systems [3]. However, despite the continued demand for e-monitoring systems to track policy and program roll out, there is little research describing the development, use and adaptation of information technology systems, generally [4], or for prevention, specifically. There is also no in-depth analysis of how these systems sit within the design of larger on-going processes to increase uptake of evidence-based programs within a complex system of practice with its diverse accountabilities.

Meanwhile, in clinical settings, the use of e-monitoring systems to record delivery of services has not created expected gains in patient outcomes [5]. In the main, electronic systems designed to increase evidence-based practice have not worked the way they were first supposed. For example, inefficiencies in practice have resulted because workflows have been restructured and roles renegotiated [6–8]. Given this track record, Greenhalgh and colleagues [6] conducted a systematic review using the meta-narrative technique to synthesise evidence on electronic patient records. They identified nine different perspectives on what the act of e-monitoring means or represents, as encapsulated by different research traditions. This ranged from studies of the impact of e-monitoring technologies on patient outcomes to investigations of how relationships and practices change because of the e-monitoring. They recommended that future studies explore the ‘hidden’ and collaborative work of staff and ‘how staff contextualize and prioritize [different types of] knowledge for shared use’ [6]. Notably, only 12 of the 94 studies they reviewed used ethnographic techniques. Greenhalgh and Swinglehurst [9] subsequently recommended ethnography to further knowledge about how e-monitoring implementation technologies are situated in practice.

In the field of preventive policy and program implementation some large-scale e-monitoring systems have been abandoned due to poor design and incongruity with practice [10, 11]. Other researchers have documented how e-monitoring systems have been so bespoke that they became irrelevant at the end of projects, and not transferred to other contexts [3]. This is interesting because health promotion has a history of initiative-taking and innovation in evaluation [12] and thought leadership in quality improvement [13]. And while Maycock and Hall [14] cautioned against the type of performance monitoring which stifles innovation and locks health promotion too much into current approaches to problems, they also called on the field to be proactive in designing new ways forward in practice improvement. This is to ensure that any new implementation, performance and quality management processes are consistent with the philosophy and practice of health promotion, rather than falling passively into imposed ‘business paradigms’ [14].

Within this broader context, we report a study of Australia’s first large-scale e-monitoring system to facilitate the dissemination of evidence-based obesity prevention programs into every primary school and childcare centre across the state of New South Wales (NSW). In contrast to the somewhat disappointing history of e-monitoring systems we have summarised so far, the Population Health Information Management System (PHIMS) has been used state-wide since 2014 and continues to adapt. Following recommendations from Greenhalgh and Swinglehurst [9], we use ethnographic methods. We outline a study protocol to investigate how an e-monitoring system aids obesity prevention program implementation. In particular, the research is intended to be attuned to the diversity of multi-level contexts within which action takes place, to appreciate how context may shape both program implementation and the use of the e-monitoring system [9].

## Context

In NSW, health promotion teams in local health districts (LHDs) have been in place since the late 1970s. The role of the teams is to promote and support the physical, mental and social health of all residents in the district, and to create supportive environments for health. On top of this basic level of responsibility and accountability, funds occasionally become available to expand capacity in particular priority domains (e.g. tobacco, falls prevention and nutrition).

In 2008, the National Partnership Agreement on Preventative Health between the Commonwealth Government and the States launched a large-scale effort to deliver setting-based programs to address the increasing prevalence of chronic disease [15]. Three flagship programs of the Healthy Children Initiative (HCI), Go4Fun, Live Life Well at School (LLW@S) and *Munch & Move*, were scaled up for state-wide delivery [16]. The latter two programs aim to improve primary school environments and early childhood education and care service environments by supporting healthy eating and physical activity [17, 18]. The evidence base on which these programs were designed was established, in part, by a previously implemented at-scale suite of childhood obesity interventions that demonstrated a 1% per year reduction in childhood obesity, against an otherwise increasing prevalence [17, 18].

LLW@S and *Munch & Move* are delivered at a local level by purposively funded, dedicated HCI positions in health promotion units across all 15 LHDs in NSW. Each LHD receives funds from the NSW Ministry of Health to implement these programs and to reach specified targets for each. The delivery approach involves HCI teams supporting primary schools and services to achieve a number of specified, evidence-based practices aimed at organisational changes to improve food and physical activity environments in those settings. The achievement of these practices is monitored through key performance indicator (KPI) targets defined by the Ministry of Health and written into the overarching service level agreements between the Ministry of Health and the LHDs [19, 20]. The KPIs are implementation targets. That is, the achievement of specified practices and policies (such as water provision instead of sugary drinks and physical activity breaks) represent a fully implemented program which should logically contribute to obesity reduction. The two programs are delivered to all primary schools and centre-based child care services in NSW. Currently, 91% (3320) of child care services and 83% (2126) of all primary schools are participating. The delivery cost is estimated to be $1500 per site per year.

The Population Health Information Management System (PHIMS) is an electronic, web-based monitoring system, purpose-built and designed via a collaboration with the Ministry of Health, the NSW Office of Preventive Health and LHD representatives. The two overarching purposes of the system are to (1) assist health promotion practitioners with local HCI program delivery of two programs, LLW@S and *Munch & Move*, and (2) record the achievement of practices which inform KPI reporting and quality improvement. At the LHD level, health promotion practitioners use PHIMS to support the day-to-day work of program delivery by tracking and planning visits to sites to provide support, documenting their interactions with sites, and recording site-level progress towards KPI achievement (e.g. sites’ achievement of healthy eating and physical activity practices). Data about progress towards KPI achievement is available for LHDs and the Ministry of Health to review in real time, providing up-to-date information about the status of each LHD in relation to their performance against KPIs. The data is used to monitor the quality and extent of program implementation and to inform service improvements. The additional investment in the design and implementation of PHIMS was approximately $2.5 m over two financial years and approximately $400K annually for support and maintenance.

PHIMS is based on the principle of user-centred design [21] and is consistent with a ‘tight-loose-tight’ approach to using it for policy and program implementation [22]. That is, agreements between the parties are tight (specific) about the problem and goal, loose (flexible) about how to reach the goal, and tight (specific) about the target to be achieved [22]. This is largely consistent with recommendations from Plsek and Wilson [23] to health care managers that they should recognise health care settings as complex adaptive systems and that they should act consistently with this theory for quality improvement purposes. That is, to amplify feedback about system performance; to provide minimal specifications when introducing change, but maximum information about purpose, goal, possibilities and choices; to build structures to encourage interaction/sharing about new actions taken and ideas tested; and to detect and communicate success/failure rapidly.

## Methods

### Study design

**Fig. 1:**
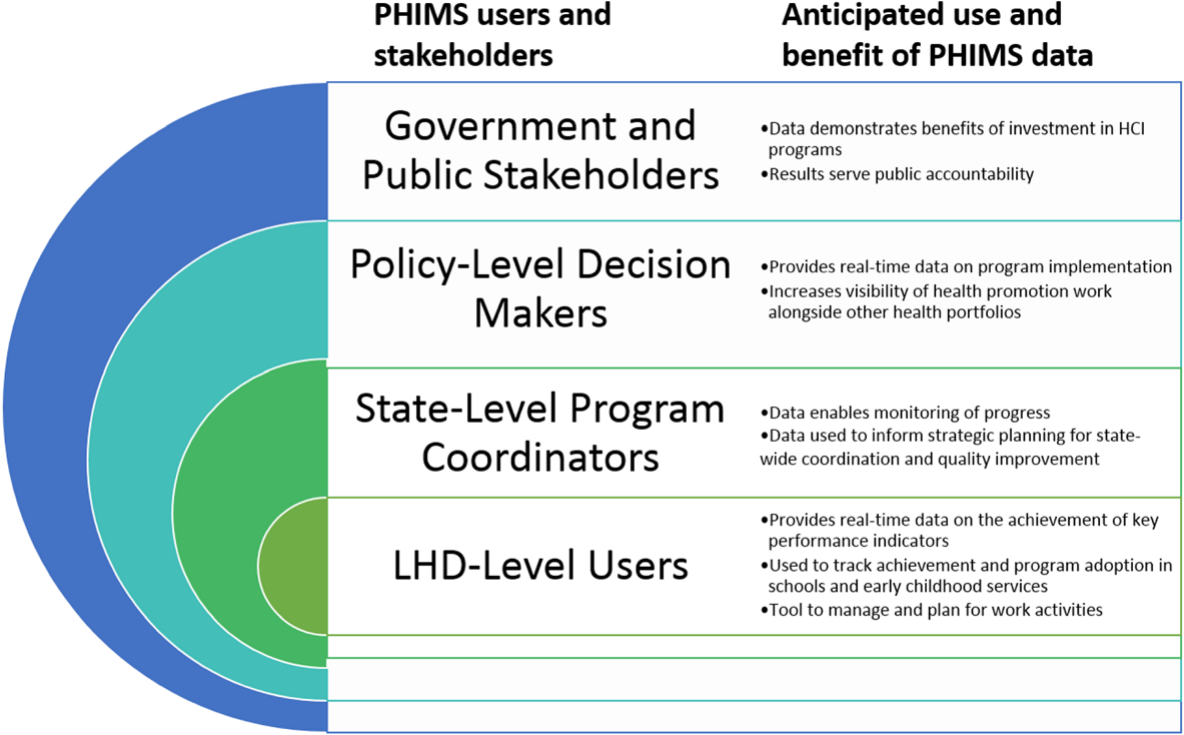
Levels of PHIMS stakeholders and designers’ anticipated use of PHIMS data

This is a multi-site case study in every LHD of NSW (*n* = 15), taking a mixed-methods approach to examining the use of PHIMS to aid the delivery of HCI programs. PHIMS operates at numerous levels of the NSW Health system. We expect the meaning and value of PHIMS, and the role of performance monitoring more generally, to vary according to the differential understanding of how users at different levels interpret the role of PHIMS (see Fig. 1). We adopt an ethnographic approach by which we may understand the multiplicity of users and sites from LHDs to policy-level decision makers. In addition, we will undertake key informant interviews, participatory workshops for data interpretation and an on-line social network analysis of the collaborative relationships within and across LHD health promotion teams.

### Research questions

The primary overarching research question is: How does PHIMS intersect with health promotion practice? We will examine how this is manifest in specific phenomena such as how PHIMS is used in health promotion practice, how PHIMS has shaped health promotion practice, how PHIMS has been embedded into practice, and how much practice is represented in PHIMS. Essentially, we are studying the programs’ scale up through the lens of the recording system. We are also interested in capturing the ‘ripple effects’ of having PHIMS data newly available for use among health policy decision makers.

### Research objectives

1. Describe the diversity of teams and contexts within which PHIMS is used and the differences in use, if any
2. Observe the breadth and intensity of work that goes into supporting early childhood services and schools to adopt practices and how this translates to data in PHIMS
3. Explore factors that influence adoption of practices within early childhood services and schools and the extent to which they are captured in PHIMS
4. Examine how PHIMS sits alongside other methods to structure, organise, record and manage health promotion practice at the local level
5. Understand how the technology interacts with the process of practice and how roles, routines and activities are impacted and how data are used
6. Articulate what matters most to health promotion practitioners in their practice—e.g. their values, attitudes and actions
7. Identify how performance monitoring via PHIMS use has impacted the field of health promotion in NSW from myriad perspectives including, for example, practitioners, health promotion managers, state-level program coordinators and funders, partnering sectors, and policy-level decision makers

Note that ongoing surveillance by NSW Health is tracking target achievement in obesity prevention program roll out (i.e. whether the KPIs about program adoption in schools and childcare are being met) and obesity prevalence rates.

### Sites and sample

This study is conducted with HCI teams across the state of NSW, the Office of Preventive Health who manages HCI at the state level, and policy-level decision makers and administrators of the PHIMS data system located within the Ministry of Health. HCI teams are situated within health promotion units in 15 LHDs that comprise the health promotion workforce across NSW. We also anticipate collecting insights from other health promotion practitioners in the LHDs (within which the HCI teams are situated) to answer more general questions about the context of practice. Because each LHD operates independently, the staffing structure for each unit varies with some staff exclusively appointed to HCI and others with split appointments. The number of staff on each HCI team range from 3 to 25 people.

### Data collection and analysis

Data collection will occur in four phases. The design of the instruments and data collection approach in each phase is informed by findings from previous phases. Four data collection activities are planned: (1) ethnography with local and state-level HCI teams; (2) semi-structured interviews with policy-level decision makers who play a role in preventive health activities, and/or whose work is informed, in part, by PHIMS data; (3) workshops with health promotion practitioners during which we present data from the previous two phases to validate findings and, finally, (4) a social network analysis with LHD health promotion units. Analysis will occur on an iterative and ongoing basis and findings will be shared with the partnership team to gain deeper insights into the study questions, refine the study approach for subsequent phases, and feedback to inform quality improvement processes in the delivery of HCI programs.

The study is described to LHDs as a way to understand what happens in practice in rolling out the HCI programs and using PHIMS to plan and record implementation and achievement of the KPIs. The image of an iceberg is used in communicating study objectives and in explaining that the research team would like to understand what goes on ‘beneath the surface’—i.e. what is not being seen and/or counted by PHIMS—and how PHIMS fits into everyday practice. Fieldwork is expected to occupy 12 months.

#### Ethnographic observations

The purpose of the ethnographies (2–3 days in each LHD) is to observe staff’s day-to-day practice and use of PHIMS at the LHD level, and at the state coordinators’ level, aiming for moderate participation [24]. Field notes will be taken documenting staff interaction with PHIMS, including the frequency and duration of use and the reasons for use, how PHIMS is talked about between staff and other types of monitoring tools that staff use to guide practice and collect data. Additionally, we will document the activities that staff engage in to deliver the HCI programs, and collect data on the collaborative nature of practice within the health promotion office, looking specifically at interactions of the HCI staff to other teams in the health promotion units. This information will be used to provide contextual information about the culture and organisation of each LHD to help us better understand variations in PHIMS usage and acceptance across HCI teams.

When possible, we will observe interactions between LHDs. Observing inter-LHD interactions will help to identify what aspects of PHIMS use and embeddedness are common and generalizable, and which are specific to individual sites. Examples of inter-LHD interactions may include monthly conference calls between supervisors, Health Promotion Officer working groups, workshops and whole-of-state meetings. As much as possible, inter-LHD activities will be identified in advance (e.g. whole-of-state meetings, monthly conference calls); however, others will be identified on an ad hoc basis, in collaboration with the Ministry of Health, the Office of Preventive Health and the LHDs. As the field work progresses, we will review findings to identify concepts or questions that require further examination.

The field-based research team comprises three researchers. Each will keep detailed and descriptive field notes of each site visit, observation, meeting and discussion [25]. Interviews will be audiotaped and transcribed. Each researcher will also maintain reflective field notes on the process of conducting this research. When appropriate, we will audio-record observed events (e.g. meetings, discussions with staff) to support the field notes. Researchers will use these recordings to verify accuracy of their field notes but they will not be transcribed verbatim unless required.

#### Interviews

We will conduct semi-structured interviews [26] with policy-level users of PHIMS data to explore different users’ perspectives of the impact of PHIMS use on health promotion practice. Interviews with policy makers will occur after ethnographies, and development of the interview guide will be informed by the initial findings. As opportunities arise during field visits, we will engage participants in conversational interviews [27] and/or follow-up with individual participants via telephone interviews to clarify or expand on observations of activities in the field. These interviews will be audio-recorded and transcribed verbatim.

#### Group workshops

The research team will present initial findings on what we have observed to the LHD teams and to the directors of those teams, and engage them in interpretation and discussion, in keeping with a participatory approach [28]. The purpose of the workshops is to validate preliminary findings, provide an opportunity for LHDs to address or correct any misunderstandings, and to enable the participants take part in the interpretation of our findings. Workshops provide an opportunity for further observation and understanding of how results are understood, translated, qualified and challenged. Data collected from workshops will include audio-recordings, notes made by researchers, and workshop materials (e.g. written feedback) that may be produced in the context of the interpretation process.

#### Additional data sources

We will also collect documents and reports, including, for example, policies, organizational charts for health promotion teams and LHDs, and evaluation summaries that will further inform our understanding of the organization of teams and processes, the local culture of practice, and strategic thinking about the implementation of HCI programs.

#### Analysis of qualitative data

The qualitative analysis will be an iterative and ongoing process, occurring during and after each phase is completed with each LHD so that findings from each LHD are used to inform the data collection approach in subsequent LHDs [29]. For the initial analysis, we will use a grounded theory approach to generate a project codebook. First, the existing field notes will be read sequentially, in full by four team members, to obtain a sense of the context and progression of activities over time. Then, each field note will be read line-by-line with the researcher highlighting each key concept and writing a note, impression or initial analysis about each [30, 31]. Next, the researcher will begin grouping similar codes together that reflect the same concept. Each researcher will do this process independently. The researchers will then meet to share initial codes and groupings. As a group, we will discuss the coding, develop definitions and coding criteria and create the initial codebook. During this stage, each data source will be coded by at least two researchers. We aim to keep codes and groupings of codes initially generally broad to enable more detailed analysis in later stages [32]. Subsequent coding will use the initially developed codebook, or derive new and more specific codes depending on the specific study question.

All coding will occur on an ongoing basis, as data is generated from the field, using NVivo [33] to facilitate organization and retrieval of codes for the next stage of analysis. The team will meet regularly to review the codes and their application and to refine the codebook as needed. During the coding process, we will mark meaningful stories for later retrieval and analysis using narrative techniques to identify cause-and-consequence thinking and illustrate key practice values [34]. To ensure replicability, we apply triangulation of investigators as well as triangulation of methods [35]. We also endeavour to ensure that the ‘voice’ in the research [36] is clearly identifiable as the researchers’ and that readers are given enough information about the researchers’ choices and interpretations to judge if the argument he/she makes is valid [36]. We are not able to offer direct member checking [35] as a means to get the informants’ view of our data, due to the anonymity required for the partnership model. But we have chosen participatory research methods [28] to gain feedback on our representation of perspectives and to involve participants in data interpretation [37].

Following initial coding, we will create LHD data summaries and then a cross-site typology to describe variation in teams and their engagement with PHIMS, HCI implementation practices and KPI achievement. The adequacy of the typology will be assessed using five criteria (based on Hunt [38]): Is the phenomenon to be classified adequately specified? Is the classification characteristic adequately specified? Are the categories mutually exclusive? Is the typology collectively exhaustive? And finally, is the typology useful?

#### Social network analysis

Social network analysis (SNA) creates quantitative summary scores for social structures [39]. Hence, it is used to quantify how connected practitioners are with each other, how central/or isolated some players may be, and why it is easy to defuse information across some groups faster than others. In health promotion, social network analysis has been used to convey local collaborative capacity in communities by examining ties among organizations [40]. The professional networks of health promotion practitioners could be similarly associated with ease of getting things done and hence SNA is used in this study to contextualise the wider team ‘culture’ within which the HCI is being delivered and PHIMS is being used. Specifically, our interest is to describe the diversity of teams and characterise local relationship infrastructure.

SNA data will be collected via an online survey that ensures a user-friendly interface and a maximum of 10 min to complete. The key relationships to be studied will be determined through field-based consultations with HCI staff and our co-partnership team. Examples of relationships may include (a) who people turn to for information/advice/problem solving; (b) who people work with most; and (c) who people feel have similar ideas and attitudes to practice to themselves. This will be a complete network survey—that is, all the specified relationships with a bounded set of people (i.e. the local health promotion team including the HCI team). We will also investigate in general terms the amount of contact across LHDs. This question will be followed by a listing of all LHDs. This question is designed to quantify the amount of collaboration that happens, particularly among adjacent rural LHDs. We will ask SNA questions twice, to capture two time periods ‘now’ and ‘same time 12 months ago’.

Analysis of the network survey will be conducted using UCINET 6 [41]. Network graphs will be drawn using Netdraw [42]. We will calculate the density of each of the relationships. Density is the amount of ties that are present as a proportion of the total possible ties [39]. So if everyone knows each other, the density score is 100%. We will also compute the two-step reach of the HCI coordinator for each of the relationships. Two-step reach illustrates the proportion of the total number of people in the network who can be reached by a person within one link of the people who comprise his/her immediate ties. It is considered a measure of how quickly a person can mobilise resources or convey information to others. We anticipate that the differences in the SNA scores over 12 months will capture turbulence in some parts of the state (e.g. high staff turnover that potentially disrupts practice).

### Use of theory in the research

Theory will not guide our enquiry directly. Rather, it will be used as a sensitizing tool, as we identify and try to understand any universal patterns in our data. We will use theory for analysis/description and explanation, as opposed to using it for prediction or prescription [43]. This study encompasses a broad range of phenomena so no single theory can be identified as the primary means by which we will understand patterns of interest. In Table 1, we present the range of theories we anticipate will guide our understanding and analysis of the research questions. That said, our work is mainly embedded within sociological theory and an understanding of practice as a social system. For example, practitioners in the system have agency (e.g. thoughts, actions, efficacy). They appraise situations and act and adjust in ways they consider best. Their continuous actions and interactions create a social structure that shifts and responds to incentives and opportunities to act differently.

**Table 1:** Theories that will be used to analyse different domains within the research program

Aspect of the research	Theory	Focus of the theory	Use and significance
Describe the diversity of teams and practice contexts within which PHIMS is used (Objective 1)	Social network theory. Borgatti and Halgin [48]	The social structure within which a practitioner is placed may influence the way they work.	The network size, the centrality of key players and the density of ties might correspond to different PHIMS-use styles and also to the intensity of work needed to achieve targets.
Describing and understanding how the data from PHIMs on KPI achievement came into being and is used at high levels in the state bureaucracy (Objectives 2, 3, 7)	Institutional theory. Scott et al. [49]	Concerned with how the most deep and resilient aspects of social structures are created and maintained by schemas, rules, behaviours, routines.	Will be used to design interviews with high-level bureaucrats to characterise and understand the role of key actors, structures, resources and symbols in building legitimacy and authority for health promotion in an otherwise clinically dominated sector
Appreciate how PHIMS sits alongside other methods to structure, organise, record and manage health promotion practice at the local level (Objective 4)	Complex adaptive systems thinking. Axelrod and Cohen [50]	Recognises that agents in a system (practitioners) are constantly adapting to changing conditions and inventing ways to respond.	Will sensitise researchers to observe how PHIMS may be grafted onto existing self-organised structures or vice versa (‘work-arounds’). The tendency for complex adaptive systems to (constantly) reorganise could potentially be harnessed for continuous practice improvement.
Understanding how PHIMS interacts with the process of practice and how roles, routines and activities are created and how data are used (Objective 5)	Activity setting theory. O’Donnell et al. [51]	Examines the everyday settings where the dynamic interaction of people and physical objects produces regular scripts or behaviours.	Will sensitise researchers to observe the roles and symbols created by PHIMS and how practice time is impacted by PHIMS use. The theory suggests that PHIMS' embedding may be reflected in these key dimensions.
	Practice theory. Feldman and Orlikowski [52],Bourdieu [53],Gherardi [54],Gherardi [55]	Recurrent actions that create the experience of organisational reality. Describes how members of a community are socialised into a workplace or profession.	To sensitise researchers to the ways that PHIMS sits within broader ‘taken-for-granted’ ways of working, and how health promotion as a practice takes shape and is constantly renegotiated.
	Normalisation process theory. May [56],May et al. [57]	How a new organisational practice, classification, technique or artefact becomes routine. Recognises implementation as a social process of collective action.	Will sensitise researchers to specific mechanisms such as the ‘talk’ that accompanies use of PHIMS and how this represents making sense of PHIMS, creating collective collaborative work and encouraging reflexive monitoring of practice.
Articulate what matters most to health promotion practitioners—the values, attitudes and actions that most ‘define’ best practice (Objective 6)	Worldview theory. Geertz [58],Rapport and Overing [59]	A person or group's picture of how things are—self, society and the nature of things.	Will sensitise researchers to observe behaviours that demonstrate values (e.g. choice to go slowly on KPI achievement if there is an immediate gain that is valued more highly such as trust and relationship building with a school).

### Expected outputs

We will construct (1) global sketch of results at each site, including the local SNA; (2) across the LHDs, a summative global typology of how PHIMS sits within practice as well as a cross-LHD analysis of the inter-LHD social network ties; (3) an analysis in which we use qualitative insights about HCI delivery and PHIMS use to make sense of variation in KPI achievement and (4) a series of papers which answers the research questions. Further, we anticipate that through the co-production process, the Ministry of Health will use findings and learnings from this project to inform the refinement and development of future e-monitoring systems for health promotion.

### Governance and ethics

The research team is comprised of state-level administrators and policymakers from the Office of Preventive Health and the Ministry of Health, representatives from the LHDs, and university-based researchers. The research team is a part of The Australian Prevention Partnership Centre, an innovative $22.5 m partnership between the National Health and Medical Research Council, two state health departments (NSW Health and ACT Health), the Federal Department of Health and Hospitals Contribution Fund, a private health insurance agency. The Partnership Centre was established to develop stronger system-based approaches to chronic disease prevention [44]. Partnership research has long been recognized as a means to facilitate the translation of research findings into policy and practice [45, 46]. We aim to produce knowledge that is meaningful and actionable because it is co-created and relevant to the practitioners, policymakers and administrators who are responsible for the ongoing design and implementation of both HCI and PHIMS. Bi-monthly meetings held throughout the project will provide direction, feedback and insight into the findings. Synthesized, de-identified results (collected by the university-based team members) will be presented to the research partnership team to ensure anonymity of the staff and LHDs. Research ethics approval has been granted by the Royal Prince Alfred Hospital Human Research Ethics Committee (X16-0156 & LNR/16/RPAH/194), and by the research governance offices of each of the 15 LHDs.

## Discussion

Our research design offers a cross-sectional ‘snap shot’ of the way different LHDs engage with PHIMS in practice and how this relates to activities directed at achieving KPIs for obesity prevention. The degree to which researchers are invited to observe the particularities of day-to-day practice will likely differ across LHD sites. However, we anticipate that the opportunity to present in-progress findings and offer them for interpretation may open further doors if LHDs feel that they are not being fully or fairly represented. Follow-up interviews are also designed to offer further insights over time to compensate for any distortion arising from the observational period chosen. So while the initial field contact in each site can be less than a week, the relationship with each LHD extends over 12–24 months, because of ongoing involvement in interpretation.

We acknowledge that our approach does not offer a longitudinal view of how PHIMS use and integration into practice may have shifted over time. Nor does it provide an in-depth understanding of each LHD, as one might get from more time in the field at each site. This study is unique in that we are studying an e-monitoring system for implementation in preventive health that has endured. Finding out what makes an enduring system endure is important. But this does not mean that we will not be party to the types of frustrations that may have ‘killed off’ similar e-monitoring attempts elsewhere [47].

Our partnership enables access to multiple bureaucratic levels and contexts within which PHIMS is used. Such access requires an ongoing commitment to generating and maintaining trust amongst partners. This is particularly important for participants at the LHD level where some of whom, due to turnover and other contextual issues, may not feel ‘on the same page’ with the PHIMS designers and state-level data users. The research project itself offers an opportunity for some otherwise distal groups to engage in dialogue and engage in building joint understanding. The establishment and maintenance of trust is a conscious, all-partner effort. The basis of this trust is facilitated by the long history and multi-level relationships that support health promotion practice in NSW and the research approach adopted. The research partnership team also recognises (1) that e-monitoring technology is part of, but not a substitute for, larger processes of continuous quality improvement that need their own stewardship and (2) that, for some participants, obesity prevention is just one of many priorities demanding attention in the field. It is vital, therefore, that we engage in close listening and strive for continual understanding. Flexible adjustment of the research approach is also important as relationships change and as our collective understanding of practice, technology use, implementation and governance grows.

## Abbreviations

HCI: Healthy Children Initiative
KPI: Key performance indicator
LHD: Local health district
LLW@S: Live Life Well at School
NSW: New South Wales
PHIMS: Population Health Information Management System
